# Clinical Results from Vaccination With Recombinant Grass Pollen Allergens

**DOI:** 10.1080/17402520600576586

**Published:** 2006

**Authors:** Marek Jutel, Oliver Cromwell

**Affiliations:** 1 Wroclaw Medical University Wroclaw Poland; 2 Allergopharma Joachim Gazer KG Reinbek Germany


*Clinical & Developmental Immunology*, June–December 2006; 13(2–4): 389–394

Taylor & Francis

Taylor & Francis Group

### Clinical results from vaccination with recombinant grass pollen allergens

MAREK JUTEL[1], & OLIVER CROMWELL[2]

[1]*Wroclaw Medical University, Wroclaw, Poland, and* [2]*Allergopharma, Joachim Gazer KG, Reinbek, Germany*

The prevalence of allergic diseases is rapidly increasing (Bauchau and Durham 2004). Allergen-SIT, which is based on the administration of the disease eliciting allergens or derivatives, is the only treatment which leads to a life-long tolerance towards previously disease-causing allergens due to restoration of normal immunity against allergens. The treatment has been shown to be particularly beneficial in allergic rhinitis, mild and moderate asthma and insect venom hypersensitivity.

Whole aqueous extracts of natural allergen source materials such as pollens, mites, moulds and animal epithelia are the basis for therapeutic preparations that are currently used in clinical practice. The extracts are standardized in terms of total allergenic activity, or potency, and possibly the concentration of one individual major allergen, whilst product consistency is assessed in terms of protein and allergen profiles determined by various techniques including electrophoresis and immunoblotting. An extract may contain numerous proteins only some of which are allergens. The composition is determined to a large extent by the quality of the raw material and the method of extraction and purification. Raw materials are provided by certified suppliers and produced under controlled conditions, but nevertheless there are differences. In addition many extracts derived from natural materials contain endotoxin (Trivedi et al. 2003).

The use of recombinant DNA technology appears to provide a realistic means of achieving improvements aiming at obtaining of more precisely defined preparations. This technology also provides the possibility to create allergen derivatives with reduced IgE-reactivity, that is to say hypoallergenic molecules that have a reduced risk for inducing undesirable allergic reactions during the course of immunotherapy, but which retain their therapeutic activity.

Numerous allergens have now been cloned for research purposes, but as yet only a few have been developed to the stage at which they can be used in clinical studies.

### Production of recombinant allergens

Once an allergen source has been identified, messenger-RNA (mRNA) is isolated and used as a template to synthesise complementary-DNA (cDNA). IgE-immunoscreening of cDNA libraries can then be used to select those cDNA encoding allergens that most closely resemble their natural counterparts. Allergen specific monoclonal antibodies can also be used. Alternatively DNA-based screening techniques, such as polymerase chain reaction amplification, which relies on the use of oligonucleotide primers designed on the basis of known amino acid sequence data for the allergen, may be used. Phage surface display technology has also been used to great advantage to identify a number of allergens. A physical linkage between the gene product and its cDNA facilitates isolation of the specific cDNA from large libraries (Crameri et al. 1994, Crameri 1998).

The next step is to insert the cDNA into a suitable vector (viral and plasmid DNA), which enables it to be expressed and produced in a suitable host organism. Bacterial host organisms, such as *Escherichia coli*, produce the recombinant proteins without post-translational modification, and in many cases it is possible to obtain the allergens with reactivity

Correspondence: M. Jutel, Department of Internal Medicine and Allergology, PL-50-471 Wroclaw, Traugutta 57, Poland.
Tel: 48 713442164. Fax: 48 713700129. E-mail: mjutel@ak.am.wroc.pl

ISSN 1740-2522 print/ISSN 1740-2530 online © 2006 Taylor & Francis
DOI: 10.1080/17402520600576586

comparable with the native form. When correct folding and post-translational modifications, including glycosylation, become an issue then a eukaryotic expression system such as the yeast *Pichia pastoris*, baculovirus in host insect cells, and various plants including the tobacco plant *Nicotiana benthamiana* (Wagner et al. 2004) and barley (Horvath et al. 2000) may be used. However these do not necessarily introduce glycosylation that is comparable with that of the natural glycoprotein, and one example for this is the expression of the house dust mite allergen Der p 1 in *P. pastoris* which results in inappropriate hyperglycosylation (Jacquet et al. 2002).

Following a fermentation process and induction of gene expression the heterologous recombinant protein is recovered from the cell culture and purified using appropriate chromatographic methods. Optimal production conditions must be determined for each protein in order to ensure that correct and reproducible secondary and tertiary structures are achieved, but once these are established the use of modern technology enables pure protein to be produced with consistent pharmaceutical quality (Cromwell et al. 2004, Batard et al. 2005).

The so-called "wild type" recombinant allergens exhibiting IgE-reactivity comparable with that of the natural allergen are particularly suitable for diagnostic applications and may also substitute for natural allergens used in specific immunotherapy. The success of the latter is, however, dependent primarily on the T-cell reactivity and immunogenicity of the protein. The allergen Phl p 1 from Timothy grass is naturally glycosylated, but the presence of the carbohydrate moiety appears not to be important for IgE-reactivity. When compared for their ability to activate peripheral blood basophils and T-cell lines from grass pollen allergic subjects the purified natural molecule and a recombinant non-glycosylated form obtained by expression in *E. coli* gave essentially identical results (Figure 1).

The production of recombinant proteins depends initially on the creation of a master cell bank incorporating a DNA construct including the expression vector together with the cDNA encoding the protein. This is the starting point for all routine production batches. Each production run starts with an aliquot of cells derived from the one cell bank thus ensuring that the identical protein is always produced.

The quality control of the recombinant proteins is undertaken using an array of analytical methods. Identity is confirmed by reactivity with specific antibodies on immunoblot, peptide fingerprinting and *N*-terminal amino acid analysis. Purity is determined by sodium dodecyl sulfate (SDS) polyacrylamide gel electrophoresis (PAGE), native PAGE (Suck et al. 2003), both in conjunction with protein staining, size exclusion high performance liquid chromatography (HPLC) and reverse phase-HPLC, taking account of peak symmetry. Allergen concentration is expressed in terms of absolute protein concentration determined from the UV absorption at 280 nm and the specific extinction coefficient of the particular protein, and confirmed as appropriate by specific antibody immunoassays and integrated HPLC peak areas. It is important to be able to determine the potency, and with natural allergen equivalents this can be best achieved by measuring IgE-binding activity in an inhibition immunoassay. The use of such a potency measure in respect of preparations for immunotherapy assumes a relationship between IgE-reactivity and antigenicity.

Levels of possible contaminants, including endotoxin and host cell proteins, are also measured. Endotoxin (lipopolysaccharide, LPS) is derived from the outer membrane of Gram-negative bacteria and can be detected in many allergen extracts (Trivedi et al. 2003). This is a potential problem for recombinant allergens expressed in the gram-negative bacteria *E. coli*, but the chromatographic purification steps for the protein can be designed to ensure that it is rendered effectively free of contamination.

### Composition of vaccine

One concept that has propagated is that of patient tailored immunotherapy (Valenta et al. 1999). The availability of pure recombinant allergens will make it possible to perform component-resolved diagnosis and to produce IgE reactivity profiles for individual patients. However, the more immediate prospect is for the introduction of standard combinations of allergens from one source, or in some cases single allergens. One allergen or allergen derivative will suffice for an effective vaccine in cat (Fel d 1) and birch (Bet v 1) sensitized subjects. The rationale being that one major allergen dominates and accounts for most or all of the specific IgE directed against the one sensitizing agent. However, in pollen or mite sensitizations the sensitization patterns are more complex.

Grass pollen has 11 different allergens that have been identified and characterized in detail and some of these are known to occur as several different isoforms. Only some of these allergens have major general importance being associated with a high prevalence of sensitization in the grass pollen allergic population and together accounting for a large part of the specific IgE directed against the whole pollen extract.

The grass *Phleum pratense* is a member of the sub-family Pooideae, which in turn belongs to the family Poaceae. It shows very substantial cross-reactivity with other members of the sub-family (Andersson and Lidholm 2003) and can therefore, be considered as representative of grasses found in temperate regions.

The group 1 and 5 allergens are strong candidates for inclusion in a therapeutic vaccine. In excess of 90%

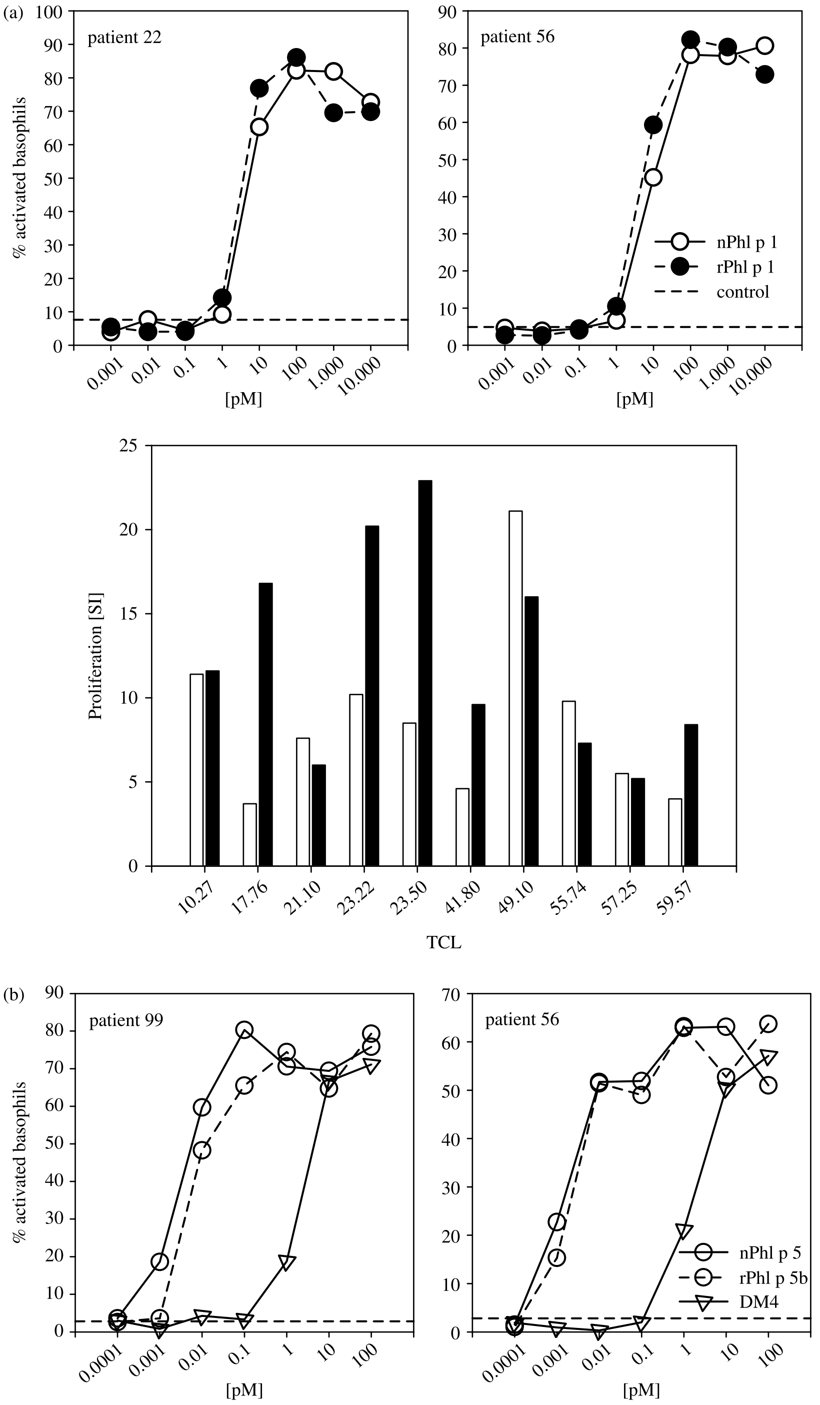

Figure 1. Comparison of natural glycosylated and recombinant non-glycosylated Phl p 1. Peripheral blood mononuclear cells from grass pollen allergic subjects were incubated with various concentrations of the allergens or the diluent solution. Basophil activation was measured by fluorescence activated flow cytometry in terms of expression of the surface marker CD203c. B. Lymphocyte proliferation, expressed as a stimulation index (SI) was determined in terms of $^{3}$H-thymidine incorporation by T-cell lines (TCL) derived from grass pollen allergic subjects. White bars natural glycosylated Phl p 1, black bars non-glycosylated recombinant Phl p 1.

of grass pollen allergic subjects react to group 1 and up to 85% to group 5 (Andersson and Lidholm 2003; Suphioglu 2000). There are two isoallergens of the Timothy grass pollen allergen Phl p 5, the principal difference between which is that two 14/15 amino acid sequences are missing from the *N*-terminal half of Phl p 5b as compared with Phl p 5a. The molecules show extensive homology, and although there is a very high degree of IgE antibody cross-reactivity, there are nevertheless some immunological differences in terms of T cell reactivity that may justify the inclusion of both isoallergens in a vaccine. The allergens Phl p 2 and 6 show prevalences of sensitization in the ranges 40–60 and 60–70%, respectively, and are thus classified as major allergens. Although as many as 80% of grass pollen sensitized subjects may have IgE antibodies directed to the group 4 allergen, the concentrations are relatively low compared with those specific for Phl p 1 and 5, as too is specific skin test reactivity. Further allergens including Phl p 7, 10, 11 and 12 are classified as minor allergens and are therefore not strong contenders for inclusion in a vaccine. Prevalence of sensitization to the calcium binding protein designated as Phl p 7 is only about 10% (Rossi et al. 2001) and subjects reacting to Phl p 10 (cytochrome c) are seldomly found (Andersson and Lidholm 2003). Both Phl p 11 (profilin) and 13 are glycosylated and a substantial part of the IgE-reactivity appears to involve cross-reactive carbohydrate determinants (CCD).

It is not realistic to represent all allergens from a particular source in the vaccine. It is reasonable to include the group 1 and 5 allergens and these may be supplemented by other relatively important allergens such as group 2 and 6, and possibly 4. A study conducted using two purified natural allergens of *P. pratense*, which were subsequently identified as Phl p 5 and 6, proved to be significantly less clinically effective than a partially purified whole allergen extract, and one can now speculate that this might have been attributable to the lack of Phl p 1 (Osterballe 1980, Osterballe et al. 1981).

### Results of clinical study

Separate aluminum hydroxide adsorbates of the recombinant allergens Phl p 1, 2, 5a, 5b and 6 were prepared and mixed together to create a therapeutic vaccine, with approximately equimolar amounts of the individual allergens. This mixture of allergens accounts for a substantial proportion of the specific IgE sensitization developed against grass pollen and, therefore, it is realistic to expect that it can be effective against a substantial part of the symptoms.

A double-blind placebo-controlled clinical trial was undertaken with 62 grass pollen allergic patients suffering from rhinoconjunctivitis with or without asthma (Jutel et al. 2005). The recombinant allergen adsorbate was administered in subcutaneous injections of increasing concentrations at 7-day intervals prior to the pollen season in 2002, starting with 0.02 μg total protein, followed by 0.16 μg and then doubling to 40 μg total protein (0.8 ml). The maximum dose contained 10 μg Phlp1, 5 μg Phlp2, 10 μg Phlp5a, 10 μg Phlp5b and 5 μg Phlp6. Maintenance injections were continued until after the subsequent pollen season with a 50% reduction during each pollen season. The median cumulative dose was 490 μg total protein, corresponding to 122.5 μg each of Phl p 1, 5a and 5b, and 61.25 μg of Phl p 2 and 6.

A symptom–medication score was the primary outcome measure to assess efficacy. Subjects kept diaries for three months over each pollen season to record nature and severity of eye, nose and chest symptoms, and type and dose of any medication. Intensity of symptoms was documented as $0 =$ no symptoms; $1 =$ mild; $2 =$ moderate and $3 =$ severe. The same rescue medication was available to all subjects and usage was scored taking account of pharmacokinetic and pharmacodynamic characteristics. A per protocol analysis included 24 active treatment and 25 placebo patients. A combined symptom–medication score adopted as primary end-point showed a 39% improvement in the active treatment group relative to placebo ($p = 0.041$). Symptoms alone improved by 37% ($p = 0.015$) and the use of symptomatic medication decreased by 36.5% relative to placebo.

A validated rhinitis quality of life questionnaire (RQLQ) was a secondary end-point (Juniper and Guyatt 1991). Questionnaires were completed every two weeks during the pollen seasons and the questionnaire following the maximum pollen count was used for analysis. Benefits were registered for those subjects on active treatment during the first pollen season. However, the per protocol evaluation during the second pollen season showed even greater differences between active and placebo treatment with an overall significant benefit ($p = 0.024$), providing further evidence of clinical efficacy. Significant effects were seen in five of seven domains tested (Figure 2). The level of clinically relevant improvement for individual subjects is considered to be 0.5, and the mean between groups differences that were judged statistically significant were all considerably in excess

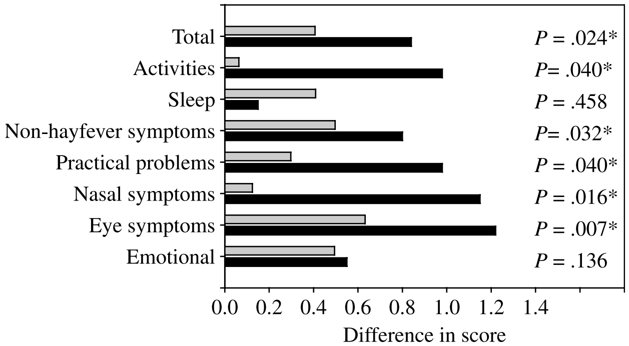

Figure 2. RQLQ for subjects undergoing immunotherapy with a mixture of five recombinant *P. pratense* pollen allergens. Bars show differences between active and placebo treatment groups in pollen seasons during the first and second years of the study, 2002 (gray) and 2003 (black), respectively. Questionnaires completed immediately following the maximum pollen count were used for analysis. Mann–Whitney *U*-test and *p* values for the 2003 season. Reprinted from *J Allergy Clin Immunol*, 116(3), Jutel M, Jaeger L, Suck R, Meyer H, Fiebig H, Cromwell O. 2005, Allergen-specific immunotherapy with recombinant grass pollen allergens, 608–613, with permission from American Academy of Allergy Asthma and Immunology.

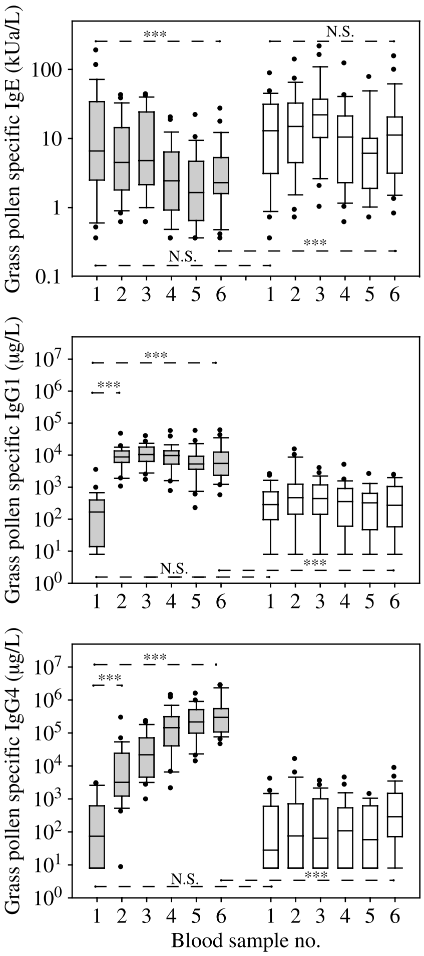

Figure 3. Grass pollen specific IgE, $IgG_1$ and $IgG_4$ antibody concentrations in blood from subjects undergoing immunotherapy with a mixture of five recombinant *P. pratense* pollen allergens. Median values with 25th/75th and 10th/90th percentiles represented by boxes and error bars, respectively, and outliers by points. Active (grey bars) and placebo (white bars) groups. Time points: (1) before immunotherapy 1/2002 to 2/2002; (2) after initial dosage increase and before pollen season 4/2002 to 5/2002; (3) after the pollen season 7/2002 to 9/2002; (4) after 12 months, 1/2003 to 3/2003; (5) before the pollen season 4/2003 to 5/2003; and (6) at the end of the study 8/2003 to 9/2003. $***p < 0.001$ and n.s., non-significant. Reprinted from *J Allergy Clin Immunol*, 116(3), Jutel M, Jaeger L, Suck R, Meyer H, Fiebig H, Cromwell O. 2005, Allergen-specific immunotherapy with recombinant grass pollen allergens, 608–613, with permission from American Academy of Allergy Asthma and Immunology.

of this figure. Conjunctival provocation tests were performed prior to therapy (inclusion criterion) and at the end of the study using a standardized 6-grass allergen extract. The concentration was increased in half-log steps starting with 5 BU/ml until a positive reaction was obtained or a maximum concentration of 5000 BU/ml (0.18 μg group 5 allergen/drop) was reached. A favorable trend was observed ($p = 0.081$) with an increase in the threshold dose in favor of treatment with the active preparation. The failure to achieve a significant result was most likely attributable to the small numbers of patients.

Active treatment induced highly significant increases in both $IgG_1$ and $IgG_4$ grass pollen specific antibody concentrations together with a significant decrease in IgE (Figure 3). $IgG_1$ increased approximately 60-fold, peaking during the first 12 months of the study. $IgG_4$ showed a continuing upward trend, achieving an approximately 4000-fold increase by the end of treatment. Specific IgE levels were not significantly different between groups at the beginning of the study, but thereafter the active treatment group showed a downward trend with values significantly less than baseline. Four subjects in each group had no Phl p 5a/b specific IgE prior to the study, but reacted to Phl p 1 and other grass pollen allergens. None of these subjects developed Phl p 5a/b IgE antibodies during the study, although the four subjects receiving active treatment developed strong $IgG_4$ and $IgG_1$ Phl p 5a/b responses consistent with induction of a protective immune response. This observation obviously needs to be substantiated and it will also be of interest to look at the prophylactic effects of the treatment in guarding against the development of new sensitizations.

Treatment related adverse events were seen in association with 78 injections (10.7%) in the active treatment group and 44 injections (5.9%) in the placebo group. Local reactions involving erythema and swelling in the vicinity of the injection site, with or without pruritus, accounted for nearly all these reactions. Single systemic reactions were seen in seven active treatment and two placebo subjects, the former including general urticaria (two cases), local urticaria (two cases), dyspnea (one case), rhinoconjunctivitis (one case) and one case of asthma two days following the injection. All these subjects continued treatment without further problems and it was concluded that the preparation showed a favorable safety when compared with findings from other immunotherapy studies.

### Conclusion

Recombinant DNA technology has delivered the prospect of a new generation of preparations for allergen specific immunotherapy. The first clinical studies with recombinant allergens have yielded very encouraging results, which suggest that there is a very good chance that such preparations will become available for use in the routine management of allergic disease.

### References

Andersson K, Lidholm J. 2003. Characteristics and immunobiology of grass pollen allergens. Int Arch Allergy Immunol 130(2):87–107.

Batard T, Didierlaurent A, Chabre H, Mothes N, Bussieres L, Bohle B, et al. 2005. Characterization of wild-type recombinant Bet v

1a as a candidate vaccine against birch pollen allergy. Int Arch Allergy Immunol 136(3):239–249.

Bauchau V, Durham SR. 2004. Prevalence and rate of diagnosis of allergic rhinitis in Europe. Eur Resp J 24(5):758–764.

Crameri R. 1998. Recombinant *Aspergillus fumigatus* allergens: From the nucleotide sequences to clinical applications. Int Arch Allergy Immunol 115(2):99–114.

Crameri R, Jaussi R, Menz G, Blaser K. 1994. Display of expression products of cDNA libraries on phage surfaces. A versatile screening for selective isolation of genes by specific gene-product/ligand interaction. Eur J Biochem 226:53–58.

Cromwell O, Suck R, Kahlert H, Nandy A, Weber B, Fiebig H. 2004. Transition of recombinant allergens from bench to clinical application. Methods 32(3):300–312.

Horvath H, Huang J, Wong O, Kohl E, Okita T, Kannangara CG, et al. 2000. The production of recombinant proteins in transgenic barley grains. Proc Natl Acad Sci USA 97(4):1914–1919.

Jacquet A, Magi M, Petry H, Bollen A. 2002. High-level expression of recombinant house dust mite allergen Der p 1 in *Pichia pastoris*. Clin Exp Allergy 32(7):1048–1053.

Juniper EF, Guyatt GH. 1991. Development and testing of a new measure of health status for clinical trials in rhinoconjunctivitis. Clin Exp Allergy 21(1):77–83.

Jutel M, Jaeger L, Suck R, Meyer H, Fiebig H, Cromwell O. 2005. Allergen-specific immunotherapy with recombinant grass pollen allergens. J Allergy Clin Immunol 116(3):608–613.

Osterballe O. 1980. Immunotherapy in hay fever with two major allergens 19, 25 and partially purified extract of Timothy grass pollen. A controlled double-blind study. *In vivo* variables, season I. Allergy 35:473–489.

Osterballe O, Lowenstein H, Prahl P, Skov P, Weeke B. 1981. Immunotherapy in hay fever with two major allergens 19, 25 and partially purified extract of Timothy grass pollen. A controlled double blind study. *In vitro* variables, season I. Allergy 36:183–199.

Rossi RE, Monasterolo G, Monasterolo S. 2001. Measurement of IgE antibodies against purified grass-pollen allergens (Ph1 p 1, 2, 3, 4, 5, 6, 7, 11 and 12) in sera of patients allergic to grass pollen. Allergy 56(12):1180–1185.

Suck R, Petersen A, Becker WM, Cromwell O. 2003. Native PAGE. 2003 is an excellent tool for investigation of natural and recombinant low molecular weight grass pollen allergens, Abstract Book EAACI 2003 58 [Suppl.74], 36. Ref. type: Abstract.

Suphioglu C. 2000. What are the important allergens in grass pollen that are linked to human allergic disease? Clin Exp Allergy 30(10):1335–1341.

Trivedi B, Valerio C, Slater JE. 2003. Endotoxin content of standardized allergen vaccines. J Allergy Clin Immunol 111(4):777–783.

Valenta R, Lidholm J, Niederberger V, Hayek B, Kraft D, Gronlund H. 1999. The recombinant allergen-based concept of component-resolved diagnostics and immunotherapy (CRD and CRIT). [68 refs.] Clin Exp Allergy 29(7):896–904.

Wagner B, Fuchs H, Adhami F, Ma Y, Scheiner O, Breiteneder H. 2004. Plant virus expression systems for transient production of recombinant allergens in *Nicotiana benthamiana*. Methods 32(3):227–234.

